# Improving health aid for a better planet: The planning, monitoring and evaluation tool (PLANET)

**DOI:** 10.7189/jogh.05.020404

**Published:** 2015-12

**Authors:** Devi Sridhar, Josip Car, Mickey Chopra, Harry Campbell, Ngaire Woods, Igor Rudan

**Affiliations:** 1 Centre for Global Health Research, The Usher Institute for Population Health Sciences and Informatics, The University of Edinburgh, Scotland, UK; 2 World Health Organization’s Collaborating Centre for Population Health Research and Training, The University of Edinburgh, UK; 3 Health Services and Outcomes Research Programme, Lee Kong Chian School of Medicine, Nanyang Technological University, Singapore; 4 Global eHealth Unit, Department of Primary Care and Public Health, Imperial College London, London, United Kingdom; 5 UNICEF, New York, USA; 6 Blavatnik School of Government, University of Oxford, Oxford, UK

## Abstract

**Background:**

International development assistance for health (DAH) quadrupled between 1990 and 2012, from US$ 5.6 billion to US$ 28.1 billion. This generates an increasing need for transparent and replicable tools that could be used to set investment priorities, monitor the distribution of funding in real time, and evaluate the impact of those investments.

**Methods:**

In this paper we present a methodology that addresses these three challenges. We call this approach PLANET, which stands for planning, monitoring and evaluation tool. Fundamentally, PLANET is based on crowdsourcing approach to obtaining information relevant to deployment of large–scale programs. Information is contributed in real time by a diverse group of participants involved in the program delivery.

**Findings:**

PLANET relies on real–time information from three levels of participants in large–scale programs: funders, managers and recipients. At each level, information is solicited to assess five key risks that are most relevant to each level of operations. The risks at the level of funders involve systematic neglect of certain areas, focus on donor’s interests over that of program recipients, ineffective co–ordination between donors, questionable mechanisms of delivery and excessive loss of funding to “middle men”. At the level of managers, the risks are corruption, lack of capacity and/or competence, lack of information and /or communication, undue avoidance of governmental structures / preference to non–governmental organizations and exclusion of local expertise. At the level of primary recipients, the risks are corruption, parallel operations / “verticalization”, misalignment with local priorities and lack of community involvement, issues with ethics, equity and/or acceptability, and low likelihood of sustainability beyond the end of the program’s implementation.

**Interpretation:**

PLANET is intended as an additional tool available to policy–makers to prioritize, monitor and evaluate large–scale development programs. In this, it should complement tools such as LiST (for health care/interventions), EQUIST (for health care/interventions) and CHNRI (for health research), which also rely on information from local experts and on local context to set priorities in a transparent, user–friendly, replicable, quantifiable and specific, algorithmic–like manner.

The last two decades have brought revolutionary changes in global health, driven by popular concern over AIDS, re–emergence of tuberculosis, novel pandemics of infectious diseases (such as SARS, H1N1pdm09 influenza and MERS CoV), the rising burden of non–communicable diseases and falling but still unacceptably high maternal and child mortality [1]. International development assistance for health (DAH) quadrupled between 1990 and 2012, from US$ 5.6 billion to US$ 28.1 billion, with the private and voluntary sectors taking on an increasing share of the commitment [2]. Influential philanthropic organizations (eg, Bill and Melinda Gates Foundation) and disease–specific public–private partnerships (eg, Global Fund to Fight AIDS, Tuberculosis and Malaria) have reformed the architecture of global health funding [3]. This generates an increasing need for transparent, fair, replicable and coordinated processes and tools that could be used to direct global health funding. The key challenges are setting investment priorities, monitoring the distribution of funding in real time, and evaluating the impact of these investments.

Currently, policy–makers have access to two types of information to assist with these three tasks. The first type is rooted in epidemiology and focuses on understanding the present burden of disease and the reduction in that burden (ie, morbidity and mortality) that a project or policy could achieve. Most recently, the ‘lives saved’ terminology has been adopted by agencies such as the Global Fund and used to drive evidence–based health policy [4]. To support this, resources have been invested (eg, by the UN agencies and the Institute for Health Metrics and Evaluation (IHME) at the University of Washington–Seattle) in generating more comprehensive and detailed estimates of global, regional and national disease burden and in getting this information into the hands of decision–makers [5]. While successful at identifying the major causes of morbidity and mortality, the focus on the burden of disease as the dominant criterion for priority setting has been criticized [6].

The second type of available information is economic and focuses largely on cost–effectiveness. Policy makers at the national and sub–national level have limited resources for scaling up cost–effective health interventions in their populations [7]. When planning the “best buys” for committing their resources in maternal and child health, they are faced with a complex task. They need to choose among at least several dozen interventions that target various diseases and vulnerable populations and decide on the most rational way to invest in the scale up of selected health interventions. Health investors usually like to know how many deaths (or episodes of disease) could be averted for a fixed level of investment. The more deaths averted per fixed investment, the more cost–effective the scale up. When the cost is low and the number of averted deaths high the intervention scale–up is highly cost–effective. When the cost is high and the number of averted deaths low then the intervention scale–up is not cost–effective. This type of analysis has been promoted by the World Bank, the Commission on Macroeconomics and Health and the recent report “Global Health 2035” [8–10].

However, this approach also has several limitations. For example, cost–effectiveness of mortality reduction does not necessarily mean that it will also be “equitable”, as these are two separate dimensions [7]. Deaths can be reduced in a highly cost–effective way when investments are targeting the wealthiest quintiles in a population, just as when they are targeting the poorest. For example, there will be instances when an equity–promoting approach, ie, trying to reach the poorest and most excluded sectors of a population with health interventions, will also be the most cost–effective approach. However, there will be instances in which this will be entirely unfeasible, and where equity–neutral or even inequity–promoting approaches may be substantially more cost–effective. In those cases, investments into health system development among the poorest that increase the quality and reduce the cost of intervention delivery may be required before intervention scale–up is planned.

While the above epidemiological and health economic approaches should, in theory, result in better–informed decisions, there may be a large gap between theory and practice. In some circumstances, sound epidemiological and health economic arguments may not result in successful project outcomes due to problems related to the mechanisms of delivery. For example, most DAH projects fail to align with the principles of the Paris Declaration and the Accra Agenda for Action, which outline best practice approaches to aid effectiveness [11].

The complexity and technocratic nature of both burden of disease and cost-effectiveness exercises have often led to these being conducted in an opaque manner and not in line with these best practice principles. These types of analyses are often unstandardized, subjective (given the huge variation in quality and type of data), time–intensive, costly and not replicable. In this article we attempt to overcome these problems by proposing a novel approach to planning, monitoring and evaluation of development assistance for health.

## PROPOSING PLANET TOOL

We present a new methodology called PLANET (PLANning, monitoring and Evaluation Tool) that could be used to improve information on the delivery and implementation of DAH. Fundamentally, PLANET is based on a combination of two useful procedures: (i) the reduction of the multi–dimensional space of a complex system to a smaller number of core variables that capture most of the variation (eg, using a statistical procedure known as principal component analysis); and (ii) the use of collective knowledge for decision–making [12,13]. Our approach brings transparency, inclusiveness, fairness and replicability to the process.

Principal component analysis is a statistical technique which reduces a very complex system of large number of variables to a small number of relatively independent “principal components” which still capture a sizeable proportion of variation in the system [13]; by defining a set of 15 “criteria”. Through this the PLANET process effectively reduces a notoriously complex and multi–dimensional task, which could be approached through an almost infinite number of “lenses”, into an exercise in which 15 of the most important (and reasonably independent) criteria for priority setting are clearly defined. If necessary these can later be weighted according to their relative importance to the users.

Collective knowledge has been increasingly recognized as a way to address these types of challenges [12]. Collective knowledge and crowdsourcing refer to the process of taking into account the collective input of a group of individuals rather than of a single expert (or small number of experts) to answer a question [12]. This is based on the observation that the average of collective judgments is closer to the truth than any single expert judgment in most circumstances [12]. The pre–requisites for this process to work are: (i) diversity of opinion (each person should have private information even if it is just an eccentric interpretation of the known facts); (ii) independence (people’s opinions are not determined by the opinions of those around them); (iii) decentralization (people are able to specialize and draw on local knowledge); and (iv) aggregation (some mechanism exists for turning private judgments into a collective decision – in this case, the PLANET method) [12]. Once each individual is given an opportunity to express their opinion in a way that is treated equally with respect to the opinion of any other individual, then the personal biases that those individuals bring into the process tend to cancel and dilute each other regardless of who the participants are. What is left is information based on the accumulated knowledge, lifetime experience and common sense of those who took part. This collective knowledge illustrates that disagreement and contest, rather than consensus and compromise, among independent minds can lead to the best decisions [12].

## CONCEPTUAL FRAMEWORK

We conceptualize DAH as a process in which multiple stakeholders invest a finite sum of money each year into improving health and development in low and middle–income countries. In theory, if the total sum was known, if it was all coordinated centrally, and if appropriate evidence on the “architecture” of missed development potential was available globally, then there would be one optimal way to invest these resources with the maximum possible impact, while all other approaches would achieve a lesser improvement in global development. In this process, the funding can be thought of the “energy” or “resource” required to fill the gaps in development, while all steps through which these funds need to be taken during this process can be seen as potentially retarding forces which may cause deviations from the most effective approach. These forces do not disappear even if more money is injected into the system. A problem is that, in reality, we neither have the detailed evidence nor the information required for the optimization of the process of DAH, nor can we monitor and centrally coordinate the flows of funding.

**Figure 1:**
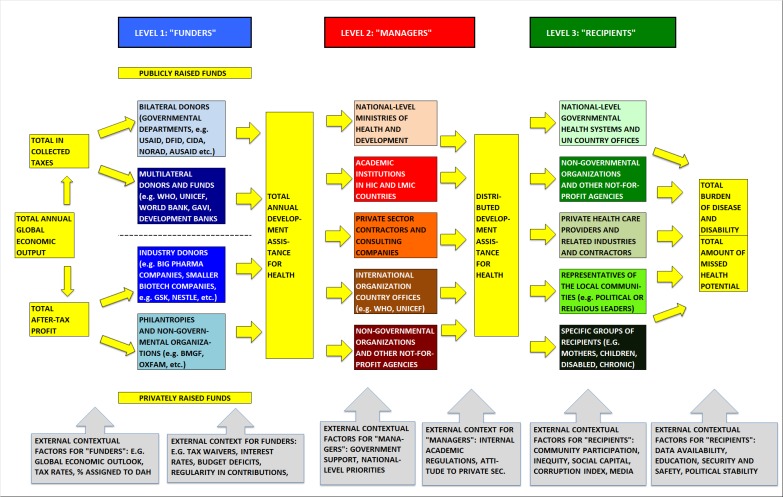
A summarized overview of the structure and some key determinants of function of the global development assistance system.

However, regardless of that, we can develop a conceptual framework that can systematically define all the fundamentally important retarding forces that are at work through this process, and try to assess, for each initiative (based on the collective knowledge of the persons most closely informed about each step in the process), how likely it is to complete its mission, and how vulnerable it is to retarding forces (**Figure 1**).

Building on McCoy et al 2009 [14], we identify three functions associated with DAH and the associated stakeholders. The first function is labeled ‘providing’ and is concerned with the need to raise or generate funds (the funders of DAH) to improve global health through development. The second function is ‘managing’ and is concerned with the management or pooling of those funds, as well as with mechanisms for channeling funds to recipients (the managers of DAH). The third function is ‘spending’ and is concerned with expenditure and consumption of those funds (the recipients of DAH). It is worth noting that while this schematic establishes a clear time sequence of the key events in the DAH process, several actors work across all three levels simultaneously. Nevertheless, similar to McCoy et al. 2009 [14], we believe that these categories provide a useful framework for studying the DAH process.

## FUNDERS OF DEVELOPMENT ASSISTANCE FOR HEALTH GRANTS

**Figure 2:**
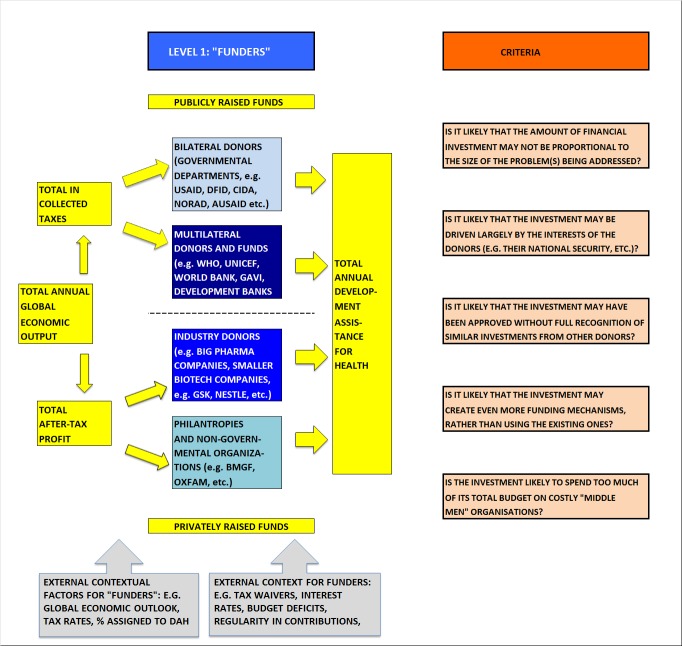
The level of funders and key performance risks at this level.

The first level of stakeholders of interest are the funders of DAH, referred to here as donors, which could include philanthropists, government or international organizations, and the investors from the private sector and industry. Donors have become increasingly aware of the importance of measuring success in terms of political sustainability but have not been in possession of a clear framework or technology to help them undertake this task effectively. Often their priority is on disbursing resources according to internal interests, or they find delivery data too difficult to collect accurately, or too politically sensitive (**Figure 2**).

At the level of donors, several factors could hinder the effectiveness of investments. First, donors could misalign the size of their support (financial commitment) with the size of the problem (burden of disease). An unprecedented amount of money is being pledged and used to fund health services throughout the world. However, several studies have shown that funding does not correspond closely to burden [2]. For example, Shiffman demonstrates that within communicable diseases for the years 1996 to 2003, there were several neglected topics such as acute respiratory infections and malaria [15]. Similarly, Sridhar & Batniji noted that in 2005, funding per death varied widely by disease area from US$ 1029.10 for HIV/AIDS to US$ 3.21 for non–communicable disease [16]. The reasons for this misalignment could be due to the social construction of the problem [17], lobbying by vested interests [18] or the personal interests of donors [19]. *Thus, the risk that the donors are misaligning their financial commitment to a disease area with the burden it causes needs to be assessed.*

Second, donors could prioritize initiatives that focus on their national self–interest rather than those that support improved health in the recipient country. For example, since the Oslo Declaration in 2006, health and foreign policy have become increasingly linked [20]. While translating health into national security language might attract attention from high levels of government, this focus has been limited to a few high–profile problems such as AIDS, pandemic influenza and humanitarian assistance and not expanded to less glamorous areas such as health systems, malnutrition or water and sanitation [21]. In fact a review of six countries’ policies illustrates that most strategies tend to be catalyzed and supported by concern with surveillance and control of infectious disease [22]. *Thus, the risk that a development project serves national self–interests, such as economic, geopolitical or security, rather than improved health outcomes in the recipient country needs to be established.*

Third, donors could fail to coordinate their activities. The current architecture of funding of global health and development is characterized by fragmentation, lack of coordination and even confusion as a diverse array of well–funded and well–meaning initiatives which descend with good intentions on countries in the developing world [23]. However ambitious or well–intentioned these initiatives might be, it becomes difficult in this environment for recipient governments to develop and implement sound national plans for their country. While there is, in general, little incentive for various development partners to coordinate their activities, some development projects work better through a joint strategy. *Thus, the risk that development partners will fail to coordinate their activities for a specific project needs to be established.*

Fourth, donors could invest in new players and models rather than strengthening and building on the existing institutional infrastructure. As noted above, there has been a continuous expansion in the number as well as type of actors involved in DAH. Instead of examining how the existing institutional infrastructure –specifically the WHO and World Bank – can be reformed to deliver on projects, new initiatives are launched that attempt to compensate for their shortcomings [24]. For example, the World Bank has an important role to play in DAH given its long history working in countries through governments, as well as in its knowledge–bank role. Similarly the WHO is unique in being governed by 193 member states and its role in setting evidence–based norms on technical and policy matters, highlighting best practices that improve health globally and monitoring and coordinating action. *Thus, the risk that a development project will result in a new institution rather than working through the existing institutional infrastructure needs to be established.*

Finally, donors could fund their initiatives in a way that results in too much funding going to more costly institutions. As McCoy et al. discuss, global health is a multi–billion dollar industry, and there are clearly competing interests amongst different actors to make use of this funding [14]. For example, pharmaceutical companies appear to benefit considerably from global health programs that emphasize the delivery of medical commodities and treatments. NGOs, global health research institutions and UN bureaucracies also have an interest in increasing or maintaining their level of income and thus tend to prefer that funding from major donors flows through them (as managers of funding), rather than directly to developing countries. Further scrutiny is needed on aid flows in global health to assess whether they are being captured by vested interests and used to support inappropriate spending on the private commercial sector or on a large and costly global health bureaucracy and technocracy. *Thus, the risk that a development project will be designed in a way that results in too much funding going to costly organizations needs to be established.*

## MANAGERS OF DEVELOPMENT ASSISTANCE FOR HEALTH GRANTS

**Figure 3:**
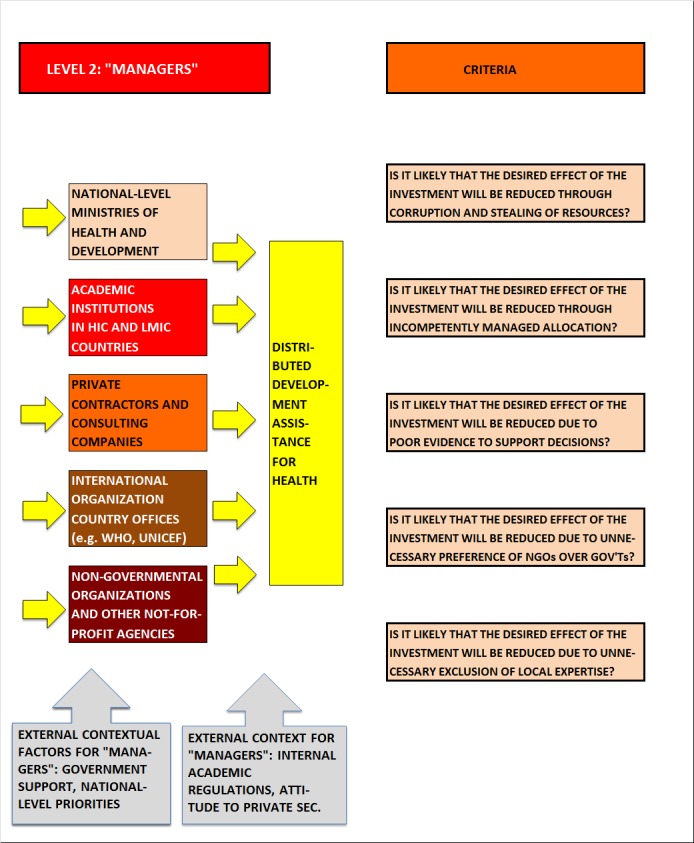
The level of managers and key performance risks at this level.

The second level of stakeholders in DAH consists of the managers of DAH grants. These could be national government ministries, NGOs, academic institutions in donor or recipient countries, private sector (with pharmaceutical companies and biotech industries), various private or not–for–profit independent consultants and country offices of international organizations. Managers are often torn between global priorities, specifically the priorities of donors, and being accountable to local communities and the ultimate recipients of aid (**Figure 3**).

At the middle level, several factors can hinder the effectiveness of investments. First, managers could deliberately steal resources from the investment for their own benefit, ie, the risk of corruption. The need to identify and address corruption and weak governance is often lost in the commitment to raise funds and expand services [25]. *Thus, the risk that funding from the project will be stolen needs to be assessed.*

Second, managers could inadvertently channel resources to purposes other than project objectives because of miscommunication, lack of competence, or lack of capacity [26]. For example, those managing the project may not have the necessary technical or administrative skills to meet key objectives. *Thus, the risk that managers inadvertently channel resources to purposes other than project objectives due to lack of competence needs to be assessed.*

Third, managers could lack credible information and evidence to maximize the cost–effectiveness of investments. The basis of cost–effectiveness is that interventions should not only have established effectiveness in reducing disease burden but also represent an effective use of resources. For a certain budget, population health would then be maximized through choosing interventions that show the best value for money. Most information about cost–effectiveness, such as that generated through the WHO–CHOICE project, are available at the regional level [27]. This creates challenges when applying these estimates to country and district level projects. *Thus, the risk that managers lack good information on the cost–effectiveness of investments needs to be assessed.*

Fourth, managers could route funding through non–governmental organizations or private sector bodies rather than working through governments. In the past two decades there has been a move towards funding non–state actors, especially by the newer funding institutions [23]. For example, the Global Fund’s use of country–coordinating mechanisms gives a larger voice to civil society as it is supposed to include a wide range of actors in a participatory process. The US government, particularly through its HIV/AIDS funding, predominantly funds faith–based organizations and NGOs. The marginal involvement of developing country governments in many DAH projects raises questions about long–term sustainability [28]. However, in some situations funding through NGOs or private sector bodies rather than through governments can work better but this should be carefully considered over a long term time horizon. *Therefore, the risk that a project routes funding through nongovernmental organizations or private sector bodies rather than through government needs to be assessed.*

Fifth, managers could exclude the participation of local experts and the inclusion of local evidence in the processes of priority setting. Managers face strong incentives to orient ‘upwards’ towards the donors that are funding the project [29]. They have little incentive to include local experts and local knowledge. *Thus the risk that local experts and local evidence are excluded in the processes of priority setting needs to be assessed.*

The above are the first ten PLANET criteria to evaluate an initiative on DAH. The informants for these aspects would include policy–makers in various global health institutions as well as health economic, governance and health systems experts (**Table 1**).

**Table 1:** Questionnaire for the implementation of PLANET

Level	Planning	Monitoring	Evaluating
**1 – Donors**	1. Is it likely that the amount of financial investment may not be proportional to the size of the problem(s) being addressed? 2. Is it likely that the investment may be driven largely by the interests of the donors? 3. Is it likely that the investment may have been approved without full recognition of similar investments from other donors? 4. Is it likely that investment may create even more funding mechanisms rather than using existing ones? 5. Is the investment likely to spend too much of its total budget on costly ‘middle men’ organizations?	1. Is the amount of financial investment disproportional to the size of the problem(s) being addressed? 2. Is the investment driven largely by the interests of the donors? 3. Is the investment being implemented without full recognition of similar investments from other donors? 4. Is the investment creating even more funding mechanisms rather than using existing ones? 5. Is the investment spending too much of its total budget on costly ‘middle men’ organizations?	1. Was the amount of financial investment disproportional to the size of the problem(s) being addressed? 2. Was the investment driven largely by the interests of the donors? 3. Was the investment approved without full recognition of similar investments from other donors? 4. Did the investment create even more funding mechanisms rather than using existing ones? 5. Did the investment spend too much of its total budget on costly ‘middle men’ organizations?
**2 – Managers**	1. Is it likely that the desired effect of the investment will be reduced through corruption and stealing of resources? 2. Is it likely that the desired effect of the investment will be reduced through incompetently managed allocation? 3. Is it likely that the desired effect of the investment will be reduced due to poor evidence to support decisions? 4. Is it likely that the desired effect of the investment will be reduced due to unnecessary preference for NGOs over government? 5. Is it likely that the desired effect of the investment will be reduced due to unnecessary exclusion of local expertise?	1. Is the desired effect of the investment being reduced through corruption and stealing of resources? 2. Is the desired effect of the investment being reduced through incompetently managed allocation? 3. Is the desired effect of the investment being reduced due to poor evidence to support decisions? 4. Is the desired effect of the investment being reduced due to unnecessary preference for NGOs over government? 5. Is the desired effect of the investment being reduced due to unnecessary exclusion of local expertise?	1. Was the desired effect of the investment reduced through corruption and stealing of resources? 2. Was the desired effect of the investment reduced through incompetently managed allocation? 3. Was the desired effect of the investment reduced due to poor evidence to support decisions? 4. Was the desired effect of the investment reduced due to unnecessary preference for NGOs over government? 5. Was the desired effect of the investment reduced due to unnecessary exclusion of local expertise?
**3 – Recipients**	1. Is it likely that the desired effect of the investment will be reduced through corruption and stealing of resources? 2. Is it likely that the investment may unnecessarily create parallel local implementation structures? 3. Is it likely that the investment may not be well aligned with local priorities or fail to involve local communities? 4. Is it likely that the investment may seem unethical, inequitable, or in any other way unacceptable to recipients? 5. Is it likely that the desired effect of the investment will be reduced due to lack of adequately trained human resources?	1. Is the desired effect of the investment being reduced through corruption and stealing of resources? 2. Is the investment unnecessarily creating parallel local implementation structures? 3. Is the investment not well aligned with local priorities or failing to involve local communities? 4. Is the investment unethical, inequitable, or in any other way unacceptable to recipients? 5. Is the desired effect of the investment being reduced due to lack of adequately trained human resources?	1. Was the desired effect of the investment reduced through corruption and stealing of resources? 2. Did the investment unnecessarily create parallel local implementation structures? 3. Was the investment misaligned with local priorities or did it fail to involve local communities? 4. Was the investment unethical, inequitable, or in any other way unacceptable to recipients? 5. Was the desired effect of the investment reduced due to lack of adequately trained human resources?

## RECIPIENTS OF DEVELOPMENT ASSISTANCE FOR HEALTH GRANTS

**Figure 4:**
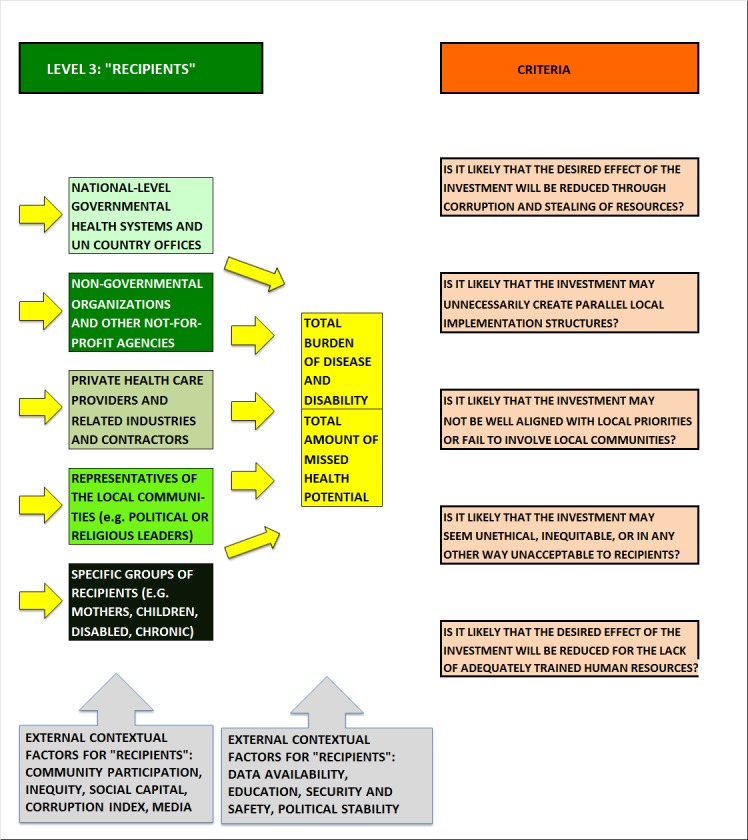
The level of recipients and key performance risks at this level.

The third level of stakeholders includes all those involved in the final stage of DAH of reaching the recipients (ie, government health systems, NGOs, private health care providers, local community representatives, and recipient groups (eg, mothers and children) themselves, including the operational workforce. At this level, several factors could hinder the effectiveness of investments (**Figure 4**).

First, the primary recipient could deliberately steal funding or commodities from this process for his/her own benefit. Numerous studies have documented such problems, for example, in the procurement of health supplies, in under–the–table payments for services, and in nurses and doctors who fail to show up at their clinics but nonetheless collect their salaries [30]. *Thus, the risk that funding from the project will be stolen needs to be assessed.*

Second, the recipient could set up unnecessary parallel structures to deliver on the project rather than working through government or ‘horizontally’. Horizontal interventions are defined as those that strengthen the heath care system, improve health systems service and delivery, and address general non–disease specific problems such as health worker shortages and stock outs of medicines and supplies [31]. Despite the consensus that DAH should be funded horizontally, most financing is channeled vertically (defined as setting up separate systems to deliver on the objectives often related to specific diseases). In recent years much of the funding has been directed to address HIV/AIDS, malaria and TB [2]. The imperative to show measurable results in a short–time frame results in setting in place short–term fixes that deliver on the project with the problem that relatively little funding may go towards capacity–building or working through government. *Thus the risk that a project will result in unjustified parallel local implementation structures rather than work through the existing health system needs to be assessed.*

Third, the project may not be aligned with local priorities or promote community involvement. The choice of a DAH priority directly affects recipients’ health, meaning that these individuals should also have the right to participate in deciding on the priorities and implementation of the project [32]. If this participation is to be meaningful nationally (or locally), then the results of the participation must have the possibility of having an impact, in this case, of affecting the nature of the project. *Thus the risk that the project will not be aligned with local priorities or promote community involvement needs to be assessed.*

Fourth, the project could be seen as unethical, inequitable or unacceptable to the final recipients. In recent years policy–makers have increasingly become aware of the disparities in health status between different groups in society and the distributional impact of interventions [33]. In particular, concern focuses on the extent to which interventions reach and benefit disadvantaged groups, such as the poor, women or certain ethnicities or otherwise marginalized populations. *Thus, the risk that the project is not ethical, equitable or acceptable to the final beneficiaries needs to be assessed.*

Finally, the project may not be sustainable, defined in terms of ensuring required human resource capacity to deliver on targets and objectives. It is increasingly recognized that the success of local implementation is highly dependent on a strong health workforce [26]. Despite this awareness, much of the focus of DAH is on commodities such as vaccines and drugs. While these are of course necessary, it is people who prevent disease and administer cures. *Thus the risk that the project will lack the requisite human resources, such as trained health workers, needs to be assessed.*

The informants reporting of these final 5 criteria could be representatives of operations workforce and / or the ultimate recipients. The above factors can be used as the 15 criteria to plan an initiative on DAH at the inception stage, to monitor its implementation in real–time, and/or to evaluate previously conducted efforts. The resulting questions that could be asked of key informants are provided in **Table 1**.

## THREE APPLICATIONS OF PLANET

The PLANET approach, as defined above, has three major applications in the field of development assistance. First is in *planning* of new initiatives in development. Donors in particular might be considering different investment options and project possibilities to address problems in development. While the overarching concern is justifiably a reduction in burden of disease, running a PLANET exercise will look at other equally important dimensions that would impact on the success of the project in reducing burden of disease as well as aligning with best practice in development.

How could the framework be used? Based on this conceptual framework we have developed a questionnaire (**Table 1**) which can be used to engage three groups of respondents. These would include those with knowledge of health governance, economics and health systems as well as policy–makers intimately involved with the execution of the project. It would also include those at the local level who are likely to be involved with the delivery of the project as well as the actual beneficiaries. All relevant stakeholders would be given this questionnaire and asked to respond independently and anonymously based on their knowledge of the project. The process could be conducted by technical experts in a transparent way (eg, each vote counts equally). The outcome would be a comprehensive list of the strengths and weaknesses of particular projects against many criteria, based on the collective input of technical experts. Additional criterion or questions can be added or substituted in to ensure covering all aspects relevant to that specific project. Analysis of the respondent data would, taken together, provide a complete picture of the strengths and weaknesses of the project that would be made available publicly.

Given that donors would be running this exercise using the expertise and accumulated knowledge of respondents, an additional step is necessary. Donors would need to define the context of the exercise based on their anticipated outcomes, the population they are targeting, the time–frame they are working under as well as stating how much risk they are willing to take to reach certain outcomes. For example, the Bill & Melinda Gates Foundation might be willing to take a major risk for a high–payoff while public donors such as the UK government might be looking to minimize risk and under those conditions to maximize health outcomes. The outcome would be a comprehensive list with competing priorities ranked according to the combined scores they received in the process. Such a list would be helpful because it provides an overview of the strengths and weaknesses of competing DAH options against many criteria, based on the collective input of technical experts. The list can also be adjusted by taking the values of many stakeholders into account such as occurred during the extensive experience with the implementation of CHNRI in health research prioritization [34].

Second, PLANET can be used to monitor ongoing initiatives and receive real–time feedback on their implementation. Third, PLANET could also be used to evaluate the success of previous initiatives. Evaluation is often woefully neglected in development and efforts such as by the Center for Global Development to fill this gap have focused on the creation of new institutions with the capacity to undertake this kind of work [35]. However, no standardized methodology exists to evaluate projects across multiple criteria capturing the essence of whether or not it was successful. Furthermore, this approach is not only concerned with considerations of disease burden reductions or change in health outcomes but with the actual process of implementation of the project, its strengths and weaknesses and whether it aligns with ‘best practice.’ The implementation would be similar to that described above using a modified questionnaire (**Table 1**).

## STRATEGIES FOR DATA COLLECTION

Exploitation of collective knowledge is now possible and moreover easier and cheaper than ever before. Information /communication technology becoming a digital utility enables us now to seek input from hundreds or thousands of independent individuals at little higher cost than asking one person. We can now, in real–time, in almost every country or setting collect feedback or opinions from an estimated 6.8 billion people who actively use mobile phones (with the proportion of smartphones rapidly growing) [36]. This can be done through text–message [37,38], automated phone calls, dedicated apps, email or the internet in a device or platform agnostic manner. It is certain that this is redefining not just the norms of who provides a feedback or communication of their assessment of a programme and how and when this is done, but also how DAH and indeed health care is delivered or consumed. The PLANET questionnaire is currently being developed into an app that would be freely available to all governments, international institutions and individuals looking for a simple, tech–friendly tool to plan, monitor and evaluate DAH.

## CONCLUSION

The PLANET tool has several major advantages over existing efforts in planning, monitoring and evaluation. First, it presents a standardized methodology that can be used for planning, monitoring and evaluation of any type of DAH project, but it also has sufficient flexibility to be tailored to the context of specific projects or initiatives. PLANET would be an additional tool available to policy–makers, along with LiST (for health care/interventions) [39] and CHNRI (for health research) [13] which will involve local experts and incorporate issues of local context in the process of determining priorities in a transparent, user–friendly, replicable, quantifiable and specific, algorithm–like manner. Second, it is simple to implement and with the development of mobile–phone software, should be able to be run anywhere in the world at low–cost. The low–cost of input means it can be run multiple times resulting in real–time monitoring of DAH. Third, while respondents are protected through anonymity in feedback, the results are provided transparently. Finally, the exercise gives equal voice to all those involved in the process of development from the donor (eg, in London, Seoul or Seattle) to a manager and to a recipient (in rural Uganda, Dhaka or Antigua). The voice of local stakeholders, including operations teams and beneficiaries, is included in every exercise.

The use of these types of novel methodologies can lead to more rational planning, higher quality evaluation as well as more knowledgeable future decision–making, especially given that DAH has traditionally lacked formal tools to examine delivery and implementation. The use of such tools would promote attention to objective evidence on planning, monitoring and evaluation leading to more effective aid and ultimately better evidence on reduction in the burden of disease across the world and how this relates or could relate to specific development efforts.
